# How Age Affects Graves’ Orbitopathy—A Tertiary Center Study

**DOI:** 10.3390/jcm13010290

**Published:** 2024-01-04

**Authors:** Michael Oeverhaus, Julius Sander, Nicolai Smetana, Nikolaos E. Bechrakis, Neumann Inga, Karim Al-Ghazzawi, Ying Chen, Anja Eckstein

**Affiliations:** Department of Ophthalmology, University Hospital Essen, 45147 Essen, Germany

**Keywords:** thyroid eye disease, GO, TED, age, Graves’ orbitopathy, strabismus, reoperation

## Abstract

Purpose: Graves’ orbitopathy (GO) is an autoimmune disorder leading to inflammation, adipogenesis, and fibrosis. The severity of GO can vary widely among individuals, making it challenging to predict the natural course of the disease accurately, which is important for tailoring the treatment approach to the individual patient. The aim of this study was to compare the clinical characteristics, course, treatment, and prognosis of GO patients under 50 years with older patients. Methods: We reviewed the medical records of a random sample of 1000 patients in our GO database Essen (GODE) comprising 4260 patients at our tertiary referral center. Patients were divided into two groups: Group 1 (≤50 years) and Group 2 (>50 years). Only patients with a complete data set were included in the further statistical analysis. Results: The results showed that younger patients (n = 484) presented significantly more often with mild GO (53% vs. 33%, *p* < 0.0001), while older patients (n = 448) were more likely to experience moderate-to-severe disease (44% vs. 64%, *p* < 0.0001). Older patients showed more severe strabismus, motility, and clinical activity scores (5.9 vs. 2.3 PD/310° vs. 330° both *p* < 0.0001, CAS: 2.1 vs. 1.7, *p* = 0.001). Proptosis and occurrence of dysthyroid optic neuropathy (DON) showed no significant difference between groups (both 3%). Multiple logistic regression revealed that the need for a second step of eye muscle surgery was most strongly associated with prior decompression (OR = 0.12, 95% CI: 0.1–0.2, *p* < 0.0001) followed by orbital irradiation and age. The model showed good fitness regarding the area under the curve (AUC = 0.83). Discussion: In conclusion, younger GO patients present with milder clinical features such as a lower rate of restrictive motility disorders and less pronounced inflammatory signs. Therefore, older patients tend to need more steroids, irradiation, and lid and eye muscle surgery. Still, the risk of DON and the necessity of secondary eye muscle surgery are not or only slightly associated with age, respectively.

## 1. Introduction

Graves’ orbitopathy (GO) is an autoimmune condition, mostly associated with Graves’ disease (GD), that causes inflammation of the extraocular muscles and orbital connective tissue, leading to a progressive eye disorder with various amounts of tissue remodeling [1,2,3]. In GO, stimulating autoantibodies (TSHR Antibodies = TRAb) cause a biased activation of the TSH receptor and IGF1 receptors on orbital fibroblasts/-cytes. This induces hyaluronan production and adipogenesis and an activation of immunomodulatory cells and thus an inflammatory cascade [4,5]. Patients with GO experience soft tissue inflammation, diplopia, and proptosis to varying degrees, which can severely impact their quality of life [6,7]. In severe cases, the disease can threaten sight mainly because of optic nerve compression [8]. Currently, in Europe available treatment options can often only reduce the inflammatory symptoms, but they cannot prevent rehabilitative surgery [9,10,11]. Therefore, it is of outmost importance to reduce risk factors for deterioration and initiate anti-inflammatory treatment early before irreversible tissue remodeling happens. According to the EUGOGO (European Group on Graves Orbitopathy) 2021 guidelines, patients with moderate-to-severe, active GO are treated with immunosuppression using intravenous glucocorticoids alone or in combination with mycophenolate sodium [12]. Poor control of thyroid function and high TSH receptor antibody levels can lead to a new development of GO or the worsening of pre-existing GO [13,14,15]. Consequently, rapid achievement of euthyroidism is crucial, which is primarily performed in the case of GD with antithyroid drugs (ATDs) [14,16]. In active, mild cases, control of thyroid function and supplementation of selenium is mostly enough, and in the case of poor quality of life steroids might be considered. Often, ATDs are enough in mild cases. However, moderate-to-severe and sight-threatening cases often present with more challenging thyroid dysfunction with relapses or poor control under ATDs. In these cases, definitive treatment with radioactive iodine (RAI) ablation or thyroidectomy (Tx) is performed [9,17]. The latter is recommended in the presence of active GO stages [12]. Aside from thyroid function, several past studies have investigated factors that may contribute to individual clinical severity, including age [18,19,20], sex [1,21,22,23,24,25], and smoking [12,19]. Older, male, and smoking patients were at higher risk for more severe forms of GO. Furthermore, older GO patients presented with a different clinical phenotype comprising a more restrictive motility disorder and inflammation and less proptosis and lid retraction [26,27]. GO incidence rates show a bimodal peak in both men and women, occurring on average 5 years earlier in women than in men (40–44 vs. 45–49 years and 60–64 vs. 65–69 years) [1]. Our recent multiple logistic regression analysis of more than 4000 GO patients confirmed these results, but it indicated that younger patients are at a higher risk for moderate-to-severe and sight-threatening GO [28]. Therefore, the current study aims to further analyze this contradictory finding and compare the specific clinical features, treatment approaches, and prognoses of a cohort of GO patients diagnosed under the age of 50 with those diagnosed later in life. Finding the correlation between age at diagnosis and prognosis will help us to plan personalized treatment according to each patient’s prognosis. This is especially important in regard to the upcoming availability of new targeted therapies and their high cost and possible side effects [12,29]. To evaluate the clinical presentation and prognosis of younger and older patients, we randomly selected 1000 patients from our tertiary referral center database for a thorough work-up and consecutive descriptive analysis. 

## 2. Patients and Methods

### 2.1. Study Population

For this retrospective study, we analyzed medical records from GO patients who visited our EUGOGO (European Group on Graves’ Orbitopathy) tertiary referral center from January 2008 till December 2018. Only patients with an actual diagnosis of GO and complete data sets were included in this study. Baseline characteristics, clinical presentation, and the course of the disease (treatments, surgeries) were assessed. From this previously published database of 4260 patients (GODE: Graves’ orbitopathy Database Essen), we randomly selected 1000 patients with SPSS [29]. Baseline characteristics and the course of the disease (treatments, surgeries) were assessed based on the patients’ charts. The retrospective study was performed in accordance with the Declaration of Helsinki and approved by the Ethics Commission of the University of Essen (reference number: 22-10729-BO). All data were de-identified, and the requirement for informed consent was waived due to the retrospective nature of the study.

### 2.2. Clinical Assessment

All patients were assessed by a team comprising specialized ophthalmologists (AE, MO, YC) and a skilled orthoptist. The diagnosis of GO was established by identifying typical clinical signs during the examination, which included measurements of BCVA, slit-lamp biomicroscopy, applanation tonometry, funduscopy, Hertel exophthalmometry, assessment of subjective diplopia, and objective measurement of deviation (far and near distance) using the prism cover test and measurement of monocular excursions with Kestenbaum glasses [12,30]. Thyroid disease was categorized into Graves’ disease (active hyperthyroidism or already treated), primary hypothyroidism, and euthyroidism (no thyroid disease in follow-up examinations). In the absence of thyroid disease, we utilized clinical signs, MRI or CT images, and levels of thyroid-specific antibodies (TRAb, Anti-TPO) to diagnose euthyroid GO. The activity of GO was evaluated using the Clinical Activity Score (CAS) classification system [31,32]. A CAS score of ≥3/7 indicated active GO. Furthermore, we classified GO severity as mild, moderate-to-severe, or sight-threatening (dysthyroid optic neuropathy (DON) and/or corneal breakdown) according to the EUGOGO criteria [12]. In addition, we scored the soft tissue inflammation signs derived from CAS more gradually as follows: spontaneous retrobulbar pain (0–1), painful eye movement (0–1), upper lid edema (0–2), lower lid edema (0–2), conjunctival injection (0–1), chemosis (0–1), lid redness (0–1), and swelling of caruncle or plica (0–1). The sum builds the clinical soft tissue score (STS).

### 2.3. Statistical Evaluation

To analyze metric data, median values ($\tilde{x})$ and range or mean and standard deviation (SD) were computed. A student’s *t*-test (two-tailed) was used to assess differences between groups if the D’Agostino–Pearson omnibus normality test indicated normal distribution; otherwise, the Mann–Whitney Test was used. Fisher’s exact test was used to examine group distributions of binary variables. A linear regression was performed to determine the correlation between deviation, age, and inflammation. Multivariable logistic regression analyses were carried out to evaluate the independent relationship of significant risk factors (including age, initial vertical and horizontal deviation, and prior decompression) for the need for secondary strabismus surgery. A level of statistical significance was defined as two-tailed with 2α < 0.05. All calculations were performed using SPSS (IBM SPSS Statistics, Chicago, IL, USA, Version 22.0.0) and Graph Pad Prism (Prism 9 for Windows, Software Inc., San Diego, CA, USA, Version 9.0.0). *p*-values are provided descriptively without α-adjustment for multiple testing.

## 3. Results

### 3.1. Study Population

Of the 1000 patients randomly selected from the GODE (n = 4260 patients), 68 had to be excluded due to incomplete data sets. Thus, the analysis included a total of 932 patients with GO. Group 1 was composed of 484 patients who were 50 years old or younger (mean age 38.1 ± 9 years, range 5–50), while Group 2 was composed of 448 patients who were older than 50 years (mean age 60.4 ± 8 years, range 51–89). Both groups mainly comprised patients with GO due to GD, with similar proportions of patients treated with antithyroid drugs (ATDs) and thyroidectomy. Primary radioiodine ablation (RAI) was more often reported in the older group (34% vs. 22%, *p* < 0.0001), as well as a smoking history in the past (17% vs. 10%, *p* = 0.01). Still, currently active smokers were similarly common (47% vs. 53%, *p* = 0.12) and showed no significant differences regarding cigarettes per day. Table 1 summarizes the demographics of the study population.

### 3.2. Clinical Examination

**Figure 1:**
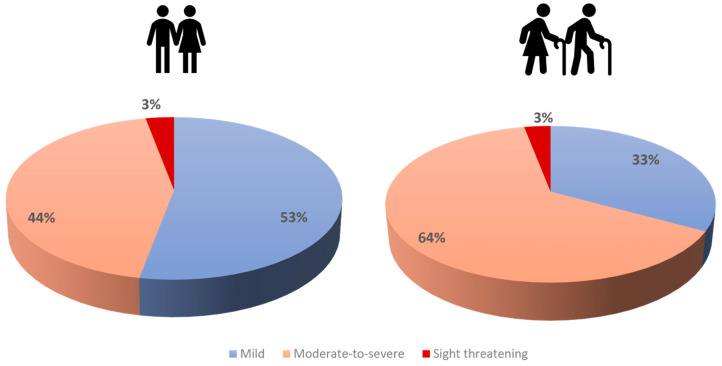
Severity of GO patients stratified by age groups (≤50 years vs. older patients).

**Figure 2:**
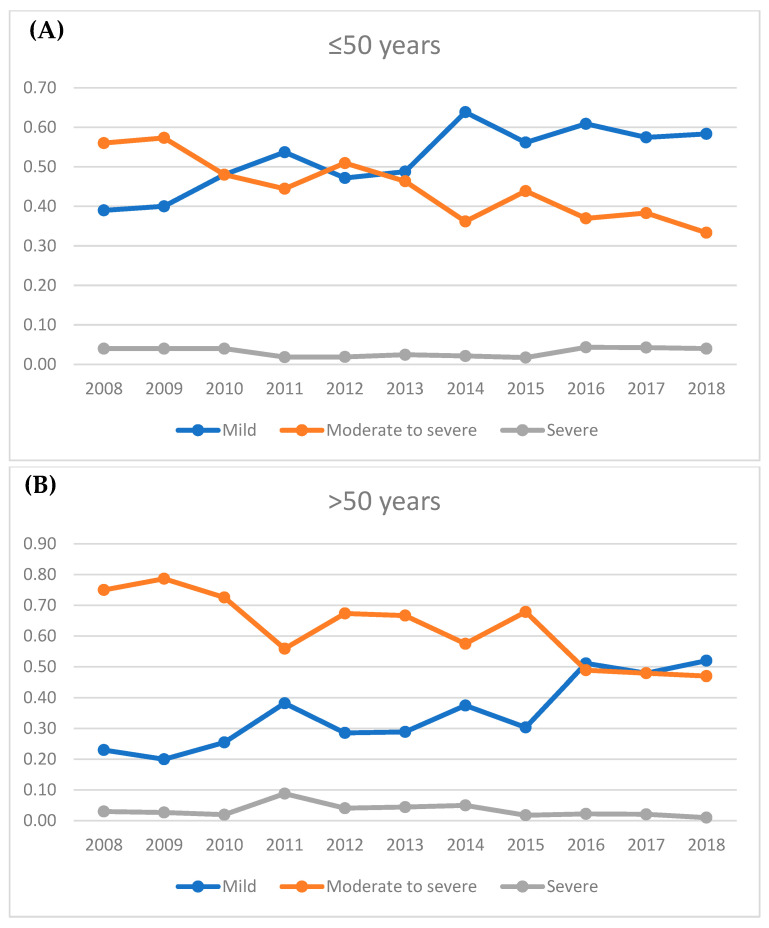
The graphs show the proportion of mild, moderate-to-severe, and sight-threatening cases for the younger (**A**) and older age (**B**) groups over the course of the ten-year analysis period.

**Figure 3:**
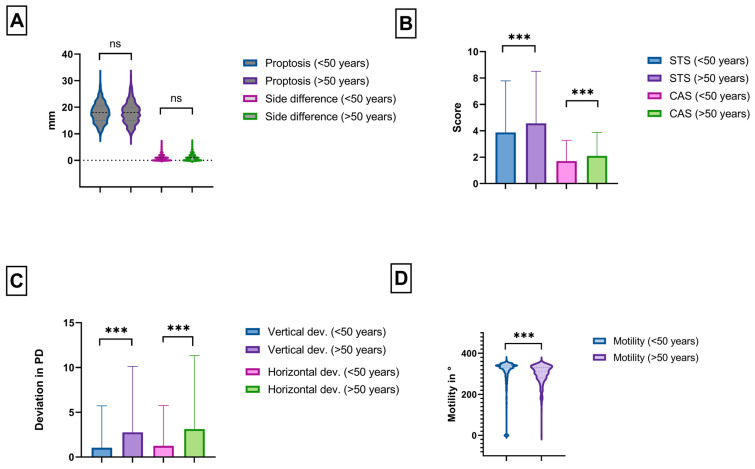
(**A**) Violin plot of proptosis and Hertel side differences for both groups. (**B**) Box plot of soft tissue score (STS) and Clinical Activity Score (CAS) for younger and older patients. (**C**) Boxplot graph of vertical and horizontal deviations for the younger and older group. (**D**) Violin plot of the overall motility of both groups. (ns = not significant; *** = *p* < 0.0011).

GO patients in the younger group presented significantly more often with mild stages than older patients (53% vs. 33%, *p* < 0.0001) and less often with moderate-to-severe GO (44% vs. 64%, *p* < 0.0001, see Figure 1). Patients with threatened sight were rare in both groups (both 3%). Thus, older patients needed steroids, orbital irradiation, and eye muscle and lid surgery significantly more often, but not orbital decompression (see Table 1). Both groups showed a trend toward a larger proportion of mild cases and a decrease of moderate-to-severe cases over the course of the ten-year analysis period (see Figure 2). This was more pronounced in the younger group, where mild cases had been the majority since 2013, with up to 64% cases of in 2014. In the older group, mild and moderate-to-severe cases had been rather outbalanced since 2016, but not with such a clear majority of mild cases as observed in the younger group. The proportion of sight-threatening cases was rather stable for both age groups. Proptosis showed no significant differences and was also not more often asymmetric in any patient group (17.9 vs. 18 mm, *p* = 0.45, see Figure 3A). Soft tissue inflammation was significantly higher in older patients (CAS: 2.1 ± 2 vs. 1.7 ± 2, *p* = 0.001; STS: 4.6 ± 4 vs. 3.9 ± 4, *p* = 0.003, see Figure 3B). Eyelid retraction was significantly more pronounced in younger patients (0.45 mm vs. 0.40 mm, *p* = 0.04). Lagophthalmos was similarly distributed between both groups (*p* = 0.78). Mean duration until first presentation in our tertiary referral center was still significantly shorter in younger patients (median 12.7 vs. 16.6 months, *p* < 0.0001), whereas older patients showed significantly longer treatment periods (15.1 ± 21 vs. 11.7 ± 17 months, *p* = 0.008).

### 3.3. Motility Disorder

**Figure 4:**
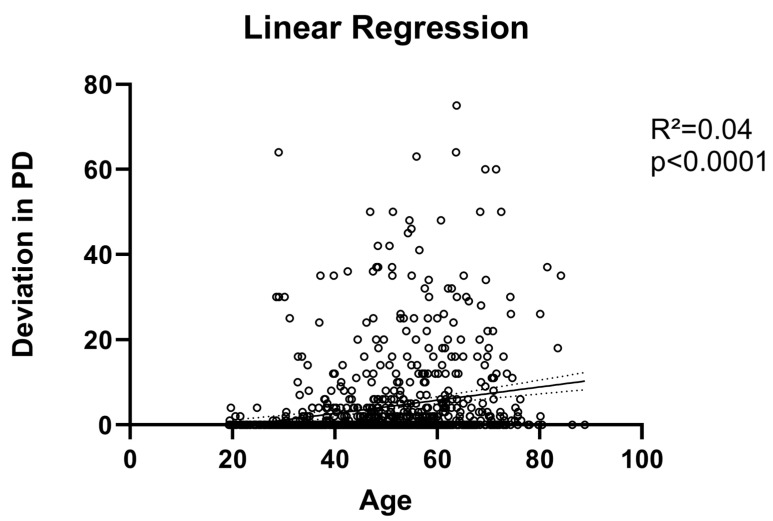
Linear regression between combined amount of vertical and horizontal deviation showed a significant correlation with age, but it could explain only 4% of the variation (R²). Data points are shown as circles, line is showing the best fit line and dotted line shows the 95% confidence interval.

**Figure 5:**
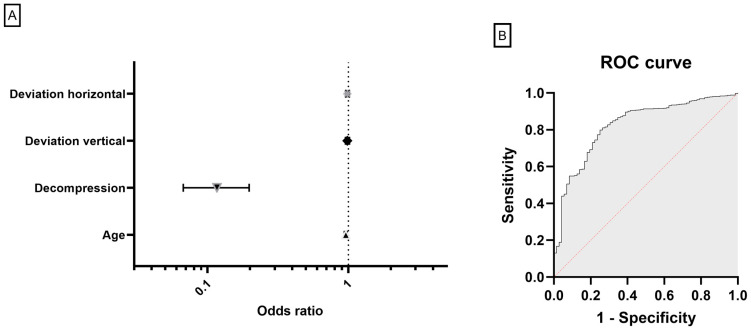
(**A**) Odd‘s ratio plot of the multiple logistic regression analyzing the correlation between the risk of strabismus reoperation and age, initial horizontal/vertical deviation, and prior decompression. (**B**) ROC curve of the same multiple logistic regression (AUC = 0.81).

Restrictive motility disorder was significantly more common in older patients: They presented with higher vertical (2.8 ± 7 vs. 1 ± 5 PD, *p* < 0.0001) and horizontal deviation (3.1 ± 8 vs. 1.3 ± 5 PD, *p* < 0.0001) accompanied by a more severely reduced overall motility (300° ± 46 vs. 304° ± 80, *p* < 0.0001, see Figure 3C,D). To further elucidate the influence of age on eye motility and strabismus, we performed a linear regression analysis. Here, we could show a significant correlation between older age and higher deviation (*p* < 0.0001, R^2^ = 0.04, see Figure 4), whereas there was no significant correlation between inflammation (CAS, STS) and deviation (*p* = 0.85 and *p* = 0.79). Since older patients not only needed eye muscle surgery more often, but also needed more procedures (0.4 ± 1 vs. 0.2 ± 1, *p* < 0.0001), we performed a multiple logistic regression to analyze the risk factors for the need for secondary eye muscle surgery: the model incorporated, aside from preoperative vertical and horizontal deviation, prior orbital decompression surgery and the age of the patients. It showed that age (OR 0.96, 95% CI: 0.94 to 0.98, *p* < 0.0001, see Figure 5) and prior orbital decompression (OR 0.11, 95% CI: 0.06 to 0.19, *p* < 0.0001) were significantly associated with higher risk for the need for secondary procedures. In contrast, preoperative horizontal (OR 0.99, 95% CI: 0.96 to 1.01, *p* = 0.38) and vertical deviation (OR 0.98, 95% CI: 0.96 to 1.02, *p* = 0.30) were not significantly associated. Multicollinearity analysis was employed to ensure the independence of the five variables, which was the case. A goodness-of fit analysis showed a Nagelkerke’s R^2^ of 0.23 and a log-likelihood ratio (G squared) of 93.9 (*p* < 0.0001) indicating a good prediction model. The area under the receiver operating characteristic curve (AUC) was observed as 0.81 (95% CI: 0.76 to 0.87, see Figure 5), indicating a good separability. The positive predictive power was observed as 92%, and the negative predictive power was observed as 20%.

## 4. Discussion

The results of the retrospective analysis of patient data from 932 patients derived from our tertiary referral center showed significant differences between younger and older patients with GO. Patients older than 50 years were more severely afflicted, especially in terms of motility. This is in line with previous reports including a combined study cohort of 503 patients. The separation of the groups according to the age of 50 was chosen since the mean age at diagnosis in most of the case series that were published was around 40 years old, and separation of groups ranged from 40 to 80 years [18,19,20,33]. Furthermore, it is known that some signs and symptoms of the disease show little change with age until passing the fifth decade [34].

### 4.1. Study Population

Interestingly, older patients were significantly more often treated with primary RAI compared to the younger patients. This is a bit surprising since in general it is not known that older patients show a lesser success rate in responding to ATDs than younger patients with GD [34,35]. Still, this might be the result of worse control of the thyroid in the older group, which could also be one of the reasons for the higher severity of GO in this group. Thus, a higher proportion of patients might have needed RAI and surgery, although the latter showed a statistically significant difference. Future studies should elucidate this matter by analyzing the course of the thyroid hormone and antibody levels in both age groups.

### 4.2. Clinical Presentation

Older patients showed significantly more moderate-to-severe disease and were more active, whereas younger patients were more often mildly afflicted. In concordance, necessary treatments also showed a clear distinction between both groups, with older patients needing more steroids and lid and eye muscle surgery. The higher prevalence of orbital irradiation in older patients might be confounded by the restrictive use of irradiation in patients < 35 years due to possible side effects. Exophthalmos and eyelid retraction are described as being the most common signs of GO in many published series [36,37]. In our patients, lid retraction was more characteristic of younger patients, whereas proptosis showed no significant differences. Consecutively, orbital decompression also showed no significant differences. Simon et al. (n = 131) also showed no difference, but a recent publication by Su et al. (n = 40) showed higher proptosis in younger patients, which might be due to the rather low sample size [26,38].

### 4.3. Severity of GO Patients over a Ten-Year Period

Our analysis showed, similar to the results of prior studies, a promising trend during the ten-year period. Similar to the PREGO studies (Presentation of Graves’ orbitopathy within European Group on Graves’ Orbitopathy centers), in recent years our cohort has shown a decrease of moderate-to-severe cases in favor of mild cases [39,40,41]. In the older group, the proportion of mild cases is comparable to the results of the PREGO study (52% here vs. 50%), whereas our younger group showed even a higher proportion of mild cases in 2018 (58%). Moderate-to-severe and sight-threatening cases cannot be compared to PREGO since they did not choose the EUGOGO classification, but they continued the distinction in moderate and severe cases [41]. In general, this shift toward more mild cases might be due to broader awareness of GO among the general public, as well as family physicians, endocrinologists, and ophthalmologists. Furthermore, anti-hyperthyroid treatments might be applied more quickly and more effectively supervised [39]. Still, this trend was much less pronounced among the older patient group. This could possibly be due to more problems in treating the underlying thyroid disease and complicating comorbidities. However, there are no similar analyses published comparing the distribution of severity stages between older and younger patients, which is why this should be further evaluated in the future.

### 4.4. Dysthyroid Optic Neuropathy

DON is one of the most serious sequelae of GO, and if left untreated, it can lead to permanent visual loss or permanent visual field loss [42,43]. In our study, DON was not more common among older patients, and the incidence was in line with previous DON reports [28,39]. In contrast, a previous study of 131 patients showed more DON in the older group without reaching statistical significance [26]. Other studies focused on DON also reported a higher risk of optic neuropathy with increasing age and hypothesized that a higher prevalence of concomitant vascular disease could be the explanation [42,44]. However, they did not report the prevalence of smoking, which our and other previous studies showed is a more important risk factor [28,45,46,47]. A recent Australian study with a large cohort of 604 patients also showed a significant correlation between age and the risk of DON. In contrast to our recent multiple logistic regression analysis of 4260 patients (GODE), their model did not include smoking or gender. These factors were, besides the thyroid disease, the strongest predictors for DON in our analysis [28]. In their model, the strongest predictors were high-grade motility disorder and strabismus. Still, since these are simply symptoms, the etiology of DON development remains unclear.

### 4.5. Motility Disorder

Regarding ocular motility, older patients showed more restriction and deviation. This is in accordance with prior publications. Furthermore, there was even a significant correlation between amount of deviation and age. To our knowledge, this is first shown by us. Interestingly, inflammation showed no correlation with deviation, which could indicate that the higher inflammation of the older group is not responsible, but rather the fact that older patients tend to have more fibrosis. Recently, a CT image review study compared muscle enlargement and motility of younger and older patients. Here, older patients were also more afflicted and showed more posterior enlargement of the extraocular muscles [38]. This might be due to higher inflammation and thus higher fibrosis in older patients. Despite this, our multiple logistic regression showed a rather low effect of age on secondary surgery. The analysis incorporated age and horizontal and vertical deviation, as well as prior orbital decompression, to analyze the risk factors for the need of secondary eye muscle surgery. Decompression was the strongest predictor, which is in accordance with previous studies, showing that medial wall decompression, especially, can lead to prolapse of the medial rectus into the ethmoid sinus and consequently to a new onset of diplopia or worsening of existing motility disorders [10,48,49,50]. Interestingly, the amount of horizontal and vertical deviation showed no significant correlation. This suggests that larger deviations can be effectively treated with combined approaches, larger recession distances, and/or tendon elongation, and only the most severely afflicted patients remain afflicted [51,52,53].Other studies looking at age differences and GO did not compare motility and deviation [20,26,27]. However, a recent study analyzing risk factors for strabismus reoperation in patients with GO showed in an analysis of 448 patients comparable results: age was not associated with the risk of undergoing a second operation, but the number of muscles initially operated on showed a strong correlation in their multiple logistic regression model (OR 1.27; 95% CI 1.03–1.59; *p* = 0.03). Unfortunately, they did not include prior decompression into their model. Still, the number of muscles should be associated with the severity of muscle involvement and could indicate in light of our results that the initial severity and complexity of the deviation (e.g., combined horizontal, vertical, and torsional deviation) is the most important. However, their study as well as ours did not include information about torsional deviation, which is why further studies are needed to support this theory.

### 4.6. Limitations

Our research should be carefully interpreted due to the retrospective nature of the study. However, have we analyzed the largest cohort of GO patients for age differences up until now, and most results are in line with previous studies. Still, our results could possibly be confounded by other factors such as genetic, ethnic, thyroid-related, and possibly further unknown associated factors. In fact, the older group showed significantly more patients who were treated with primary RAI and who were past smokers, which could partially explain the more severe forms of GO in this group. Future multi-center, prospective studies should further elucidate the relationship between age and GO.

## 5. Conclusions

We showed in our large cohort comprising 932 patients from a single tertiary referral center that there is a strong association between higher age and more severe GO and distinct phenotypic characteristics. However, we could not reproduce earlier findings of a higher risk of DON in older patients. Older patients were at a slightly higher risk of a strabismus reoperation independent of the amount of deviation, which should be considered when planning surgery. Still, prior orbital decompression was a stronger predictor.

## Figures and Tables

**Table 1:** Characteristics of study population.

	All (n = 932)	>50 (n = 448)	≤50 (n = 484)	*p*
Age at onset	49.6 ± 13	60.4 ± 8	39.2 ± 8	<0.0001 ^a^
Thyroid disease				
Graves’ disease	88%	89%	87%	0.06 ^b^
Hypothyroidism	7%	8%	8%	0.81 ^b^
Eutyhroidism	4%	4%	4%	0.62 ^b^
Thyroid treatment				
ATD	27%	23%	27%	0.102 ^b^
Thyroidectomy	39%	40%	37%	0.4583 ^b^
Primary RAI	26%	34%	22%	0.0001 ^b^
GO status at baseline				
Mild	41%	33%	53%	0.0001 ^b^
Moderate-to-severe	55%	64%	44%	0.0001 ^b^
Sight-threatening	3%	3%	3%	1.000 ^b^
Asymmetric GO	22%	24%	21%	0.2717 ^b^
Treatment period	13.3 ± 19	15.1 ± 20	11.7 ± 17	0.008 ^a^
Smoking status				
Non-smoker	36%	36%	37%	0.8650 ^b^
Smoker	50%	47%	53%	0.1201 ^b^
Past smoker	13%	17%	10%	0.0157 ^b^
Cigarettes per day	13.9 ± 9	13.8 ± 9	13.9 ± 9	0.95 ^a^
Treatments				
Steroids	49%	58%	42%	0.0001 ^b^
Orbital irradiation	29%	39%	20%	0.0001 ^b^
Lid surgery	16%	20%	12%	0.0015 ^b^
Eye muscle surgery	19%	26%	13%	0.0001 ^b^
No. of procedures	1.53 ± 0.8	0.41 ± 0.8	0.2 ± 0.5	<0.0001 ^a^.
Orbital decompression	21%	21%	20%	0.7465 ^b^

Unless otherwise stated, data are means ± SD or proportions (%) or median ($\tilde{x})$ [range]; ^a^: *t*-test/Mann–Whitney-test; ^b^: Fishers exact test.

## Data Availability

The data that support the findings of this study are available from the corresponding author, M.O., upon reasonable request.
